# Simultaneous lipid biosynthesis and recovery for oleaginous yeast *Yarrowia lipolytica*

**DOI:** 10.1186/s13068-019-1576-7

**Published:** 2019-10-08

**Authors:** Pratik Prashant Pawar, Annamma Anil Odaneth, Rajeshkumar Natwarlal Vadgama, Arvind Mallinath Lali

**Affiliations:** 1 DBT-ICT Centre for Energy Biosciences, Institute of Chemical Technology, Nathalal Parekh Marg, Matunga East, Mumbai, Maharashtra 400019 India; 2 Department of Chemical Engineering, Institute of Chemical Technology, Nathalal Parekh Marg, Matunga East, Mumbai, Maharashtra 400019 India

**Keywords:** Microbial oil, *Yarrowia lipolytica*, Continuous production, Oil capturing agent (OCA), Extractive production

## Abstract

**Background:**

Recent trends in bioprocessing have underlined the significance of lignocellulosic biomass conversions for biofuel production. These conversions demand at least 90% energy upgradation of cellulosic sugars to generate renewable drop-in biofuel precursors (H_eff_/C ~ 2). Chemical methods fail to achieve this without substantial loss of carbon; whereas, oleaginous biological systems propose a greener upgradation route by producing oil from sugars with 30% theoretical yields. However, these oleaginous systems cannot compete with the commercial volumes of vegetable oils in terms of overall oil yields and productivities. One of the significant challenges in the commercial exploitation of these microbial oils lies in the inefficient recovery of the produced oil. This issue has been addressed using highly selective oil capturing agents (OCA), which allow a concomitant microbial oil production and in situ oil recovery process.

**Results:**

Adsorbent-based oil capturing agents were employed for simultaneous in situ oil recovery in the fermentative production broths. *Yarrowia lipolytica*, a model oleaginous yeast, was milked incessantly for oil production over 380 h in a media comprising of glucose as a sole carbon and nutrient source. This was achieved by continuous online capture of extracellular oil from the aqueous media and also the cell surface, by fluidizing the fermentation broth over an adsorbent bed of oil capturing agents (OCA). A consistent oil yield of 0.33 g per g of glucose consumed, corresponding to theoretical oil yield over glucose, was achieved using this approach. While the incorporation of the OCA increased the oil content up to 89% with complete substrate consumptions, it also caused an overall process integration.

**Conclusion:**

The nondisruptive oil capture mediated by an OCA helped in accomplishing a trade-off between microbial oil production and its recovery. This strategy helped in realizing theoretically efficient sugar-to-oil bioconversions in a continuous production process. The process, therefore, endorses a sustainable production of molecular drop-in equivalents through oleaginous yeasts, representing as an absolute microbial oil factory.

## Background

Ecological concerns have called for sustainable utilization of lignocellulosic biomass while emphasizing the research for fuel replacements. The second-generation biofuels, which were introduced as blend stocks to the present-day fuels, have also ended up demanding renewable stand-in substrates for their technologies relying upon plant-based resources. These technologies have necessitated a green escalation of the highly oxygenated lignocellulosic carbon to a molecular equivalent that can functionally conform to the existing fuel infrastructure. This implies valorization of hydrogen-poor cellulosic sugars grounded at an effective hydrogen-to-carbon ratio (H_eff_/C) of 0 to more energy-dense forms, closer to the petroleum fuels (H_eff_/C = 2), through fewer, non-carbon and non-energy intensive steps of biochemical conversions [1]. To that end, the lipids, having H_eff_/C = 1.8 and specifically those produced by oleaginous organisms, project out as promising candidates. Microbial oils are, therefore, proposed as alternates to vegetable oil- or animal fat-based feedstocks which currently cater the drop-in biofuels production. These prospects are made based on anticipated microbial conversions, provided that the microbial oils also endure the productivities and market occupied by the plant-derived oils. Microbial oil production is thereby obligated for effectual carbon conversions, higher yields, productivities, and complete oil recoveries [1, 2].

The oleaginous yeasts accumulating a large amount of microbial oil under nutrient-deprived stress conditions are being screened for their oil production capacities using different carbon substrates [3, 4]. The induced stress is known to direct the carbon flux sequentially through a series of protein repressive transition phases, following the Kennedy pathway which leads to intracellular TAG accumulation in the form of lipid droplets (LD) [5]. Thus, many studies emphasize on enhancing intracellular lipid accumulation, either by increasing the Acyl-CoA pool capacity or by addressing the degradation of formed lipids [6].

The feasibility of these intracellular metabolite production processes eventually depends on the efficiency of the down-streaming for complete product recovery. Existing methodologies for intracellular oil recovery are based on energy-intense cell-disruptive techniques which account for about 70% of total production costs [7]. While, others suggest direct hydro-thermal conversions of the oleaginous cells into liquefied high energy drop-in fuel precursors [8]. These operations often affect the natural forms of intracellular lipids and further affect their oxidative stability. Moreover, the cells are also rendered unusable [9]. A judicious down-streaming is, thus, anticipated for a green and highly efficient low-cost recovery of such intracellular metabolites.

Recent studies have pointed out that the triglycerides (TAGs) formed and accumulated as lipid droplets are understood to have a more functional role rather than mere energy storage repositories when the oil production broth mainly comprises of a sole carbon source. The lipases associated with the LD membrane permit the mobility of TAGs and influence their dynamics out of the cell. While, unsaturation in the fatty acids substantially increases their fluidity [10–12]. Alteration of cell membrane permeability [13], along with the secretion of emulsifiers, has also been reported with high glucose concentrations in the fermentation media [14]. All the above responses shown by the yeast can be to maintain cellular homeostasis in an exclusive glucose solution as opposed to the limited nitrogen conditions, wherein the nitrogen scavenging mechanism prevails [5]. These symptoms in a sole carbon media demonstrate a possibility of extracellular leaching of the otherwise intracellular oil and its consequent non-destructive removal from the cell.

Despite all these reported adaptations, the low solubility of oil in water ensures that the lipids harbored through the membrane remain in close vicinity to the cells or adhere to them owing to their hydrophobicity. This phenomenon leads to the blocking of the lipid traffic across the membrane, and thus the associated fluxes through it. Besides, it also results in lower sugar assimilations, low microbial oil productivities, and ‘lipid turnover’. Attempts for increasing the extracellular oil secretion have contemplated metabolic flux manipulation through ‘push and pull’ mechanism with the addition of an organic phase in the fermentation medium [15]. Some have also shown increased oil efflux using surfactants [16]. However, these amendments are prone to considerable lipid losses and inefficient oil recoveries due to micellar entrapment. Also, the oil yields on sugar, at par with the theoretical maximum, have not been reported in any of these studies.

In previous studies using *Yarrowia lipolytica*, we highlighted a significant increase in extracellular oil production when a two-stage approach was used. Continuous biomass production in a chemostat at critical dilution rates followed by usage of cell mass for oil production in a glucose media devoid of any nutrient auxiliary ensued an augmented oil titer of 4.8 g L^−1^ [17]. Although these methods prove attractive for enhancing the extracellular oil secretion, it becomes necessary to selectively isolate the lipids from the broth as soon as they are expelled.

In this work, we study the oil production potential of *Yarrowia lipolytica* in a two-stage process and investigate the effects of addition of an adsorbent-based oil capturing agent (OCA) during fermentation. High binding capacities of the adsorbent matrices have exhibited selective isolation of organic species from the aqueous medium [18]. They have shown successful applications earlier in the purification steps [19] and also taken care of the inhibitors in fermentative processes [20]. Most of these employments were post-cell harvest and associated with the downstream processing of the fermentation broths.

Herein, we show that the simultaneous extracellular oil capture from the fermentation broth positively affects glucose assimilation by the organism and resultant oil production. The significant increase in glucose uptake rates in the presence of OCA suggests removal of adhered lipid droplets from the cell surface, allowing glucose assimilation and flux redirection to TAG synthesis. Finally, as the OCA captures oil from the cell surface and extracellular media, it is assumed to enable mobility of TAGs out of the cells facilitating higher and prolonged production of oil in the stationary phase cells. Different modes of OCA integration to the fermentation cycle have been presented to show that continuous milking of cells positively impacts lipid yields by preventing and minimizing “lipid turnover”. The inclusion of the adsorptive system with the fermenter further allows repeated cell use for extended periods, thereby improving the process economics and benignity.

## Results and discussion

### Extracellular oil production in a two-stage microbial oil production process

**Fig. 1:**
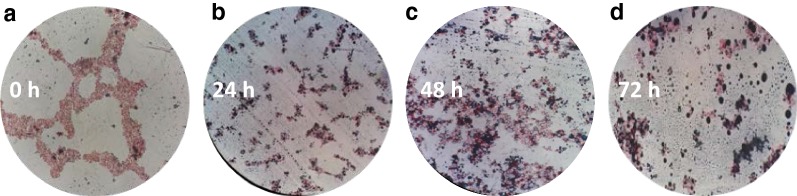
Sudan black staining of lipids produced by *Yarrowia lipolytica* in the oil production broth. The oil production broth samples collected at different time intervals after inoculation, i.e., 0 h (**a**), 24 h (**b**), 48 h (**c**) and 72 h (**d**), were stained with Sudan Black B dye, followed by counterstaining with safranin dye and then observed under oil immersion at 100× magnification. Cells appearing red, represent staining by counterstain safranin and absence of intracellular lipids (**a**). Cells stained black, represent intracellular lipid accumulation (**b**–**d**). Small black droplets (stained with Sudan Black B dye) outside the cells represent extracellular oil in the broth (**d**)

The two-stage process for oil production in *Yarrowia lipolytica* NCIM 3590, as described earlier [17], was evaluated by closely monitoring the microscopic changes in the oil production phase. Sudan black B dye was used to stain periodically withdrawn broth samples and assess their lipogenicity. Intracellular lipid accumulation was observed after 24 h (Fig. 1), it increased on the second day and further on to the third day, where a maximum number of cells appeared loaded with oil. This lysochrome stain is known for staining the intracellular neutral fats like triacylglycerols (TAG), and thus has been extensively used to gauge the intracellular lipid accumulation qualitatively. The dye diffuses through the aqueous media into the oleaginous cells, owing to its hydrophobicity and interacts with the TAGs in the form of lipid droplets, leaving the oleaginous cells stained intensely blue [21]. However, after 72 h, the broth also showed the presence of some small extracellular oil droplets stained with Sudan Black B dye. Since the dye is exclusively lipophilic and is specifically known to stain neutral lipids, the possibility of water-soluble media components getting stained is negligible.

Further insights into the two-stage system were obtained using a more selective fluorescent lysochrome, ‘Nile red’ and by contacting a hydrophobic solid-phase oil capturing agent. Nile red is known to fluoresce upon a change in medium polarity when in a hydrophobic environment due to spectral shift in excitation and emission wavelengths [22]. Moreover, the intensity of fluorescence increases with the degree of unsaturation in the lipids, thereby offering for a selective, rapid, and quantitative estimation of lipids [23]. Consequently, polar lipids like phospholipids are stained bright red, whereas the intensity and emission shift towards yellow with the increase in hydrophobicity of lipids [24]. Intracellular lipid staining is mediated by organic solvents which allow mobility of the dye inside the cells. The movement, in turn, is dependent on the concentration of the dye stain as well as the composition of the cell membrane [25, 26]. Thus, the use of a concentrated dye allowed for selectively fluorescing extracellular polar and unsaturated lipids, at higher emission wavelengths; while the intracellular/neutral lipids or TAGs appeared in a phase contrast field. Overlay of the two fields further elucidated the lipid distribution in the system.

**Fig. 2:**
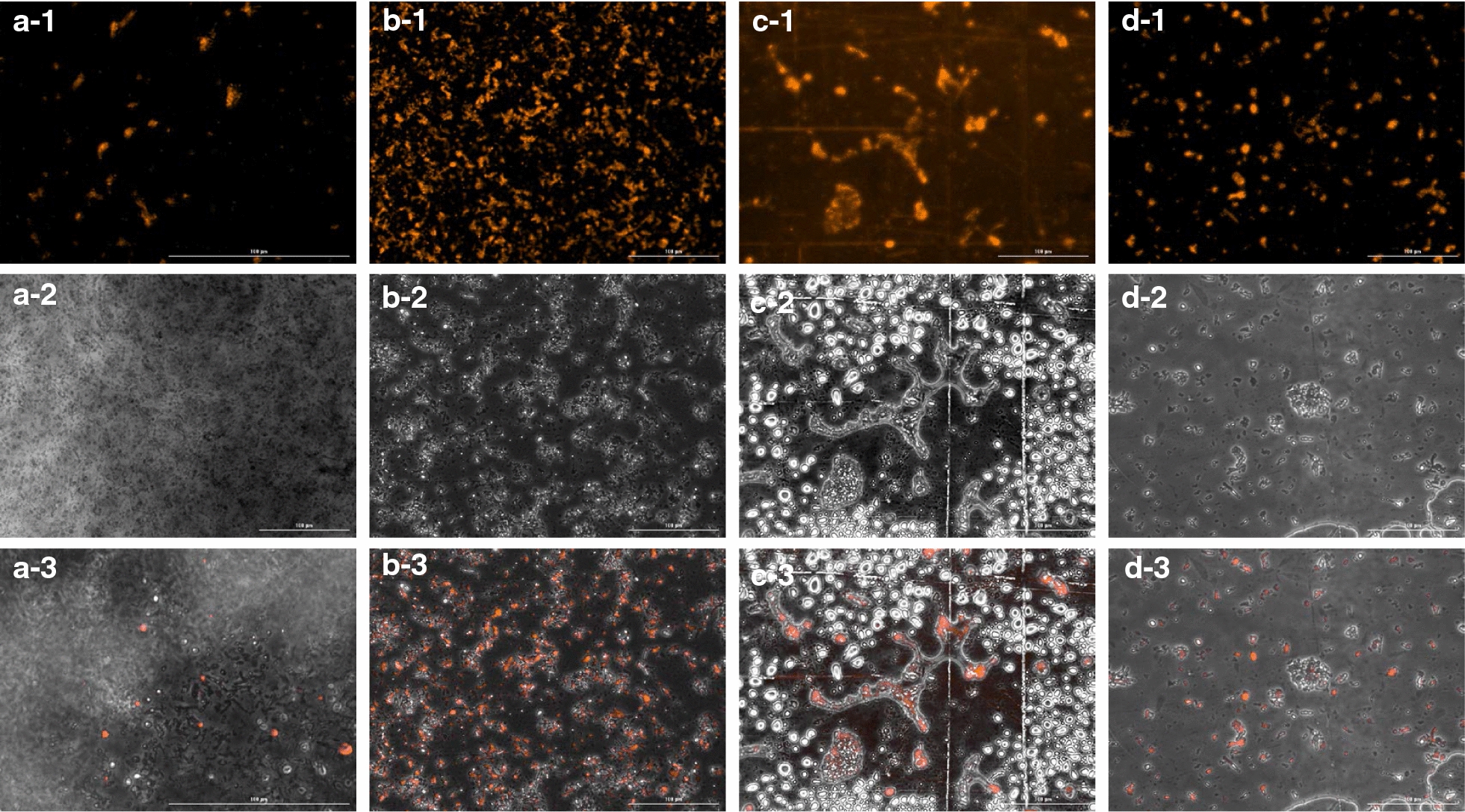
Nile red fluorescence microscopy. Broth samples with cells in the growth phase (**a**), cells in the oil production phase (**b**), cells in oil production phase with in situ OCA (**c**) and those contacted to ex situ OCA, post-oil production (**d**), were stained by Nile red dye and observed under a fluorescence microscope. Fluorescence at 593 nm shows extracellular oil in the sample (− 1); Phase contrast mode shows intracellular oil accumulation (white) inside the cells (− 2) and an overlay (− 3) shows the distribution of microbial oil in the samples

The growth phase cells, harvested from a chemostat, showed low fluorescence and marked less accumulation of intracellular lipids as well as lesser expulsion of the extracellular oil (Fig. 2). On the contrary, the cells from the oil production phase, after 72 h, showed an excellent intracellular oil production and almost an equal distribution of the extracellular oil. When the solid-phase OCA was introduced to the oil production broth, to capture the extracellular oil, the observations further elucidated the two-stage oil production process. In an in situ capture mode, wherein the OCA was added to the oil production broth at on-set of fermentation, a profound increase in the intracellular oil accumulation was seen in the cells after 72 h. There was also a considerable amount of extracellular oil in the oil production broth with in situ OCA. Whereas, in an ex situ mode, the oil production broth (after 72 h of fermentation) was contacted to the capturing agents for further 24 h. This post fermentation ex situ oil capture, thus, removed all of the extracellular oil from the aqueous broth after fermentation. The presence of intra- as well as extracellular oil in case of in situ oil capture during the fermentation, thus, implies enhancement in oil production. This enhancement in intra- and extracellular oil can be accredited to the simultaneous oil removal during fermentation.

**Fig. 3:**
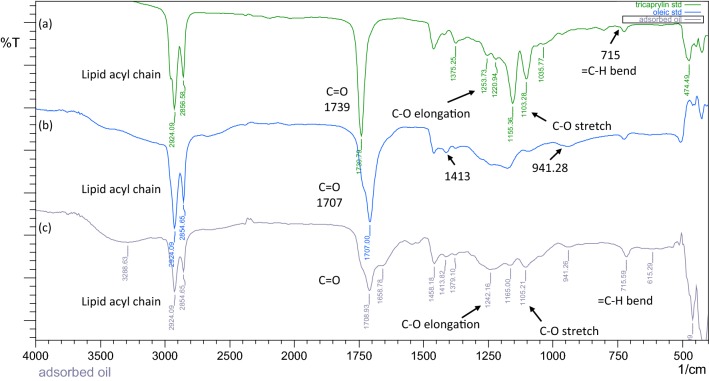
Characterization of the microbial oil captured on OCA by Fourier transform Infra-Red (FTIR) analyses. The FT IR spectra of standard triglyceride (tri-caprylin) (**a**), free fatty acid (oleic acid) (**b**), and OCA captured microbial oil (**c**), are overlaid for comparison

To ascertain this, when the same oleaginous cell samples and the OCA captured oil were characterized with FTIR, it was found that the oil production phase cells and the captured oil showed characteristic absorbance/vibrations which were specific to lipids. These were also matched with standard lipids. As seen in the Fig. 3, the captured oil showed the presence of C=O stretching band at 1708 cm^−1^, which is ascribed to fatty acids. The bands attributed to the carbon–hydrogen bond of the lipid acyl chain (–CH_2_–), asymmetric and the symmetric stretching at 2924 cm^−1^ and 2854 cm^−1^, respectively, along with (=C–H) bending vibrations at 715 cm^−1^ were observed. Moreover, the signals at 1242 cm^−1^, 1103 cm^−1^ affirming dimeric C–O elongation and C–O stretch in triglycerides were also seen in the FTIR spectra of captured oil. Additional bands at 1413 cm^−1^ as well as 941 cm^−1^ specific to angular deformations of the C–O–H bond and out-of-plane O–H, respectively, as observed in free fatty acids or dimeric fatty acids noted the traces of partial glycerides and fatty acids in captured oil [27].

**Fig. 4:**
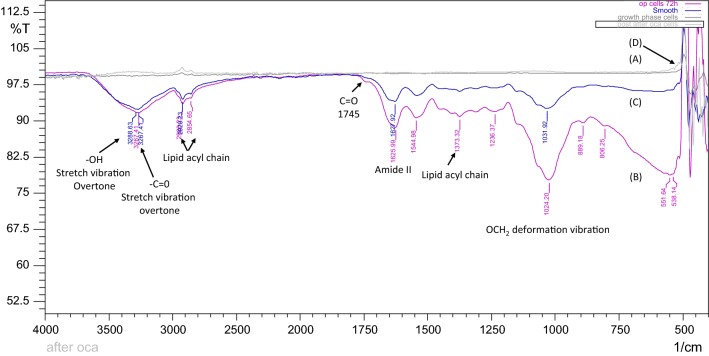
FTIR spectra of intact cells of *Yarrowia lipolytica*. Cells from the growth phase (A); oil production phase (B); oil production phase with in situ OCA (C) and those contacted to ex situ OCA, post-oil production (D) were analyzed for vibrations characteristic to the lipids

Similarly, when the intact oleaginous cells from the different phases, were subjected to FTIR analyses as described by Ami et al. [28], the resulted spectra showed similar bands to that of the lipids (Fig. 4). The C=O stretching band at 1445 cm^**−**1^ and CH_2_/CH_3_ symmetric/asymmetric acyl stretch vibrations at 2854 and 2920 cm^**−**1^ along with similar bands at 1373 and 1544 implied the presence of lipids in oil production phase cells and those cultured with in situ OCA. However, no such vibrations were observed with the growth phase cells, and the oleaginous cells contacted to ex situ OCA post-production. Furthermore, as seen in (Table 1), the intensity of vibrations was found to increase in the case of in situ OCA, validating changes in the metabolism, as observed in microscopic evaluation. This FTIR profile confirms that while the ex situ oil capture post fermentation removes the extracellular oil from the aqueous broth, the in situ oil removal during fermentation favors the oil production.

**Table 1:** Transmittance in FTIR spectra of the intact cells

Phase	Peak (cm^−1^)	Intensity (%T)	Area	Vibration
Oil production phase (without OCA)	1024.2	77.78	17.57	OCH_2_ deformation
	1625.99	88.66	4.68	C=C/asymmetric COO^−^ stretching
	2920.33	93.55	2.88	CH_2_ stretching (lipid acyl chain)
	3267.41	91.7	15.62	C=O stretching overtone
Oil production phase (with in situ OCA)	1031.92	92.6	3.65	OCH_2_ deformation
	1627.92	94.06	2.56	C=C/asymmetric COO^−^ stretching
	2920.23	94.51	2.42	CH_2_ stretching (lipid acyl chain)
	3267.41	92.38	6.74	C=O stretching overtone

Structural elucidation of the intracellular oil and the adsorbent bound oil with LC–MS showed that the intracellular oil was composed of lipids of all the types including free fatty acids, partial glycerides, triglycerides, phospholipids, etc. (Additional file 1A). However, the captured oil essentially comprised of the triglycerides or neutral fats (Additional file 1B). Since, the hydrophobic OCA show specific non-ionic interactions, they could selectively capture the neutral glycerides from the extracellular broth or the surface of oleaginous cells. Extracellular secretion of triglycerides has also been reported earlier with a strain of *Trichosporon* cultured in a media with glucose as a sole carbon source [49].

From Table 2, it can be understood that the intracellular oil accumulation increased in the oil production broth and was further amplified in presence of an in situ OCA. Moreover, the extracellular oil titer in the aqueous medium was stalled around 2 g L^**−**1^ in all the cases, except for the post-production ex situ oil capture where the OCA effectively removed all the residual extracellular oil from the broth. These studies confirmed that the in situ OCA addition had a significant effect on the metabolism and therefore on the oil production in a two-stage process and so it was decided to pursue it further and evaluate its applicability in the production process.

**Table 2:** Distribution of microbial oil in the fermentation broth

Phase	Intracellular oil in the cells (g/L)	Extracellular oil in the broth (g/L)
Growth phase	1.5	0.1
Oil production phase	3.15	2
Oil production phase with in situ OCA	5.4	2
Post-production ex situ capture of oil	2.5	0

### Two-stage microbial oil production with in situ OCA

The synchronous cells harvested after growth phase, when subjected to oil production medium, were observed to collectively contribute to lipid production as described by Warke et al. [17]. However, the problem of extracellular lipid saturation of the aqueous medium and possibly the cell membrane was understood to be the limiting factors for improvising yields in such a system. It was hypothesized that a systematic way of contemporaneous confiscation of the produced lipid from the broth would help to address the issues and result in increased oil production.

Various extractive production strategies are known for permeating the cell membrane and continuously stripping the produced oil. These techniques employ a liquid phase biocompatible extractant like long-chained alkanes [29] to increase the miscibility of the extracellular oil in the aqueous broth and thereby promote its efflux. Some also report secretion of bioemulsifiers that aid the extracellular oil. However, these alkanes can pose problems and losses in down-streaming owing to phase separation [30] or by its assimilation in yeast [31]. Recently, a novel phage-mediated permeation of the cell membrane for effective removal of the lipid was also reported [32].

Here, the use of an inert, non-miscible, hydrophobic solid-phase matrix in the form of adsorbent resins was envisaged for effective in situ capture of the produced oil from the broth. Microbial oil production was attempted after the growth phase in an oil production broth, without and with SEPABEADS™ SP70 as an in situ OCA, separately at 2-L scale in a fermenter. SEPABEADS™ SP70 is a synthetic copolymer of Styrene Di vinyl benzene (~ 850 micro-meter spherical particles), offering a high hydrophobic surface of 870 m^2^ g^−1^ for selective oil capture. Phillips et al. reported use of SEPABEADS™ SP70 for selective in situ isolation of natural products from the broth without the interference of cellular deposition/substrate adsorption [20]. Based on these aspects, the hydrophobic adsorbent of SEPABEADS™ SP70 was selected as an oil capturing agent.

**Fig. 5:**
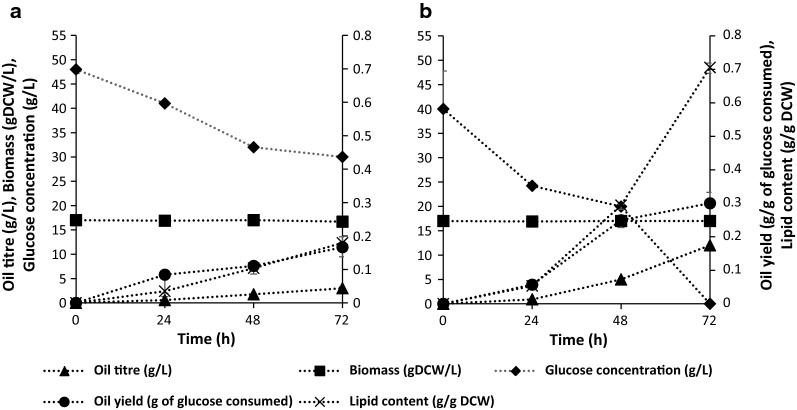
Time profiles for glucose assimilation and oil production in a two-stage production process. Oil production by *Yarrowia lipolytica* NCIM 3590 was carried out in a 2 L fermenter; **a** without and **b** with in situ OCA, to understand its effect on oil yield, oil titre, lipid content and glucose uptake

The comparative study of glucose assimilation, cell biomass viability, and oil production over 72 h revealed that the simultaneous adsorptive oil capture had the upper hand in every aspect (Fig. 5a, b).

In the batch mode of operation, when the OCA was added to the oil production broth based on their binding capacity; the dispersed beads in the aqueous broth offered a large surface area for oil adsorption and cell contact.

Total consumption of glucose was observed with an oil yield of 0.33 g g^−1^ of glucose utilized. This oil yield corresponding to the theoretical maximum yield on glucose and an oil productivity of 3.98 g L^−1^ day^−1^, were the key benchmarks achieved. Maintenance of the viable cell biomass in a stationary phase and increased total lipid content and oil titer to about 70% of dry cell weight and 12 g L^−1^, respectively, were some other added advantages along with reduced energy usage, unit operations, and associated expenses. It was interesting to note that the dry weight of the cell biomass remained constant throughout, while the total lipid content increased. This phenomenon was due to the account of extracellular oil produced in the total lipid content. Similar results were reported by Ledeshma-Amaro et al. where a metabolic flux pull for oil production resulted in lipid content of as high as 120% DCW [15].

Nevertheless, the physical effects of incorporating the resin directly into the fermentation broth in a batch process cannot always be considered as an attractive option. After a particular limit marked by complete adsorption on OCA, the oil production again ceases, necessitating the termination of the batch and replacing the OCA with a fresh substitute. Besides this, the replacement of the dispersed OCA every 72 h can itself be a cumbersome task. This approach implicitly limits the reusability of the cell biomass with a possibility of exposure to contamination.

Additionally, the sedimentation and mixing characteristics impaired the adsorptive capture large vessels. The heterogeneous solid-phase non-ionic beads can cause attrition to the contacting cells and alter the mixing characteristics in a CSTR. Also, the integrity of the OCA itself in the stirred vessel is questionable, as high shear can cause for its breakage. These concerns demand an optimization in the mode of operation to allow efficient capture of oil in the fermentation process.

### Rate of glucose uptake and oil production in a two-stage process

**Fig. 6:**
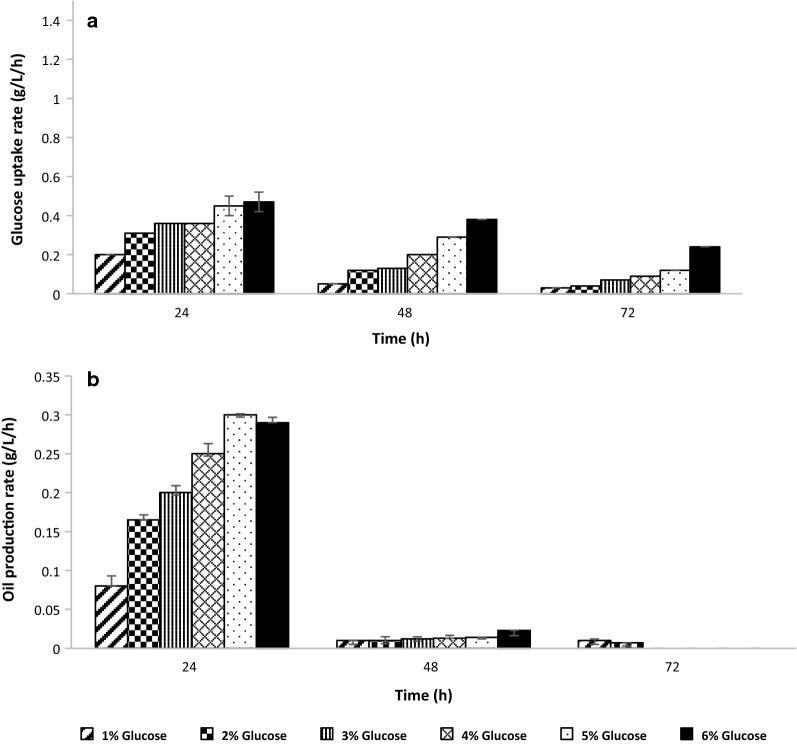
Kinetics of glucose assimilation and oil production by *Yarrowia lipolytica* in a two-stage system. The glucose uptake rate (**a**) and oil production rate/productivity (**b**) were studied over 3 days in the oil production broth with different glucose concentrations

As described in the earlier section, although the in situ OCA augmented the oil production to theoretical conversions, it was necessary to have more understanding about the oil production and substrate uptake kinetics in such an extractive production system. In this regards, the glucose uptake rate and the oil production rate were studied with different concentrations of glucose ranging from 1 to 6%, in the production phase with and without in situ OCA. It was found that the glucose uptake rate was in accordance with the oil production rate (Fig. 6a, b). It was also observed that the rate of oil production dropped with an increase in sugar concentration beyond 5% glucose concentration. As can be seen from the (Fig. 6b), there was no significant oil production after 48 h with increased sugar concentration, which denotes a saturation point for oil accumulation. Prevalence of ‘lipid turnover’ mechanism can be perceived in this case [33], as the glucose uptake rate at 6% concentration was not coherent with the oil production rate at the same concentration. Thus, at higher glucose concentrations, equilibrium was found to be attained by ex novo re-assimilation of the produced oil. This phenomenon was in nexus with oil saturation concentration of the aqueous medium and the cell surface with the lipid droplets. The effect is negligible with lower concentrations of glucose but becomes more profound, with an increase in the glucose concentration beyond the saturation limit. As a result, saturation in oil accumulation was marked by a consequent saturation in the glucose uptake, thereby signifying equilibrium in both the fluxes. 5% glucose concentration was, therefore, selected as an optimal glucose concentration for oil accumulation by *Yarrowia lipolytica* NCIM 3590, and the fermenter scale studies were carried at this concentration.

Despite being an efficient approach for directing the glucose towards oil production, the saturation effect in the ‘two-stage system’ cannot be neglected. The lower sugar utilization at high concentrations cannot be solely attributed to substrate inhibitions. Issues about product inhibition also need to be addressed while dealing with process intensification.

**Fig. 7:**
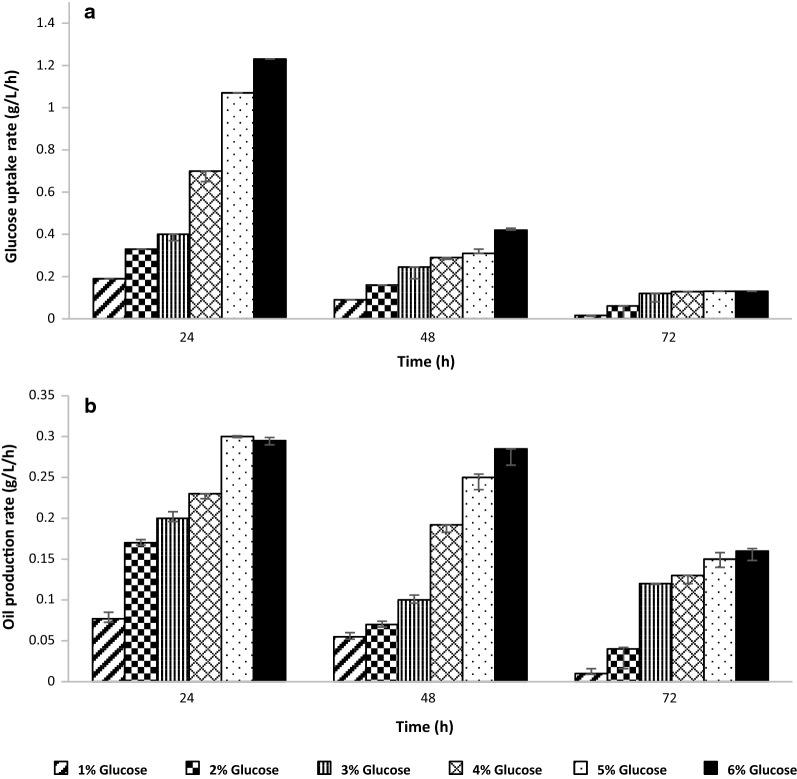
Effect of OCA addition on glucose assimilation and oil production kinetics. **a** Glucose assimilation rate and **b** oil productivity at different glucose concentrations in the presence of an oil capturing agent (OCA), was studied for 3 days

The method of simultaneous in situ oil removal from the broth was supposed to delay the oil saturation paving the way for increased lipid biosynthesis. The delayed saturation can help in maintaining a steady state or viability in the cells, with equilibrium conditions across the cell membrane. This can be achieved by an induced outflow of lipids counterbalanced to the influx of the soluble carbon source, in this case. The oil production trends and the glucose assimilation kinetics with OCA were also observed with varying initial glucose concentration throughout 72 h (Fig. 7a, b). A consensus in the trend, as observed in Fig. 6, was also seen in this case with varying concentration. However, an increased glucose assimilation and oil production were seen in this case, owing to the simultaneous removal of the produced oil. Thus, it can be concluded that the saturation was delayed and the carbon flux was pulled towards increased oil production in the presence of an OCA. The extracellular oil release and capture further averted the ‘lipid turnover’ in the oleaginous cells and delayed the attainment of ‘cellular lipid homeostasis’, which also led to the milking of oleaginous yeast cell mass for oil production.

Incorporation of the adsorbent resins in the broth, allowed for a passive increase in the glucose uptake rate, along with an increase in the oil production rate, as a timely capture of oil from the broth and cell surface, paved the way for more lipids being produced by the organism.

At lower glucose concentrations, the intrinsic mechanism of *Yarrowia* was found potent enough to deliver oil at theoretical yields without being affected by substrate or product inhibitions. This effect can be inferred from the insignificance in the glucose uptake and oil production with OCA at lower glucose concentrations (1% and 2%).

Also, it can be seen from Figs. 6, 7 that the initial glucose uptake rate for the first 24 h corresponded the oil production rate only up to 50 g L^−1^ glucose concentration. With further increase in glucose concentration, there was no significant increase in oil production, which denotes the inherent substrate uptake and oil production capacity of an organism. Thus, although the oil production with OCA was always higher to its counterpart even at these higher glucose concentrations, the inherent capabilities cannot be ignored. Hence, 50 g L^−1^ glucose concentration was considered optimal, and all the operations were designed accordingly.

### Continuous production and on-line oil capture

**Fig. 8:**
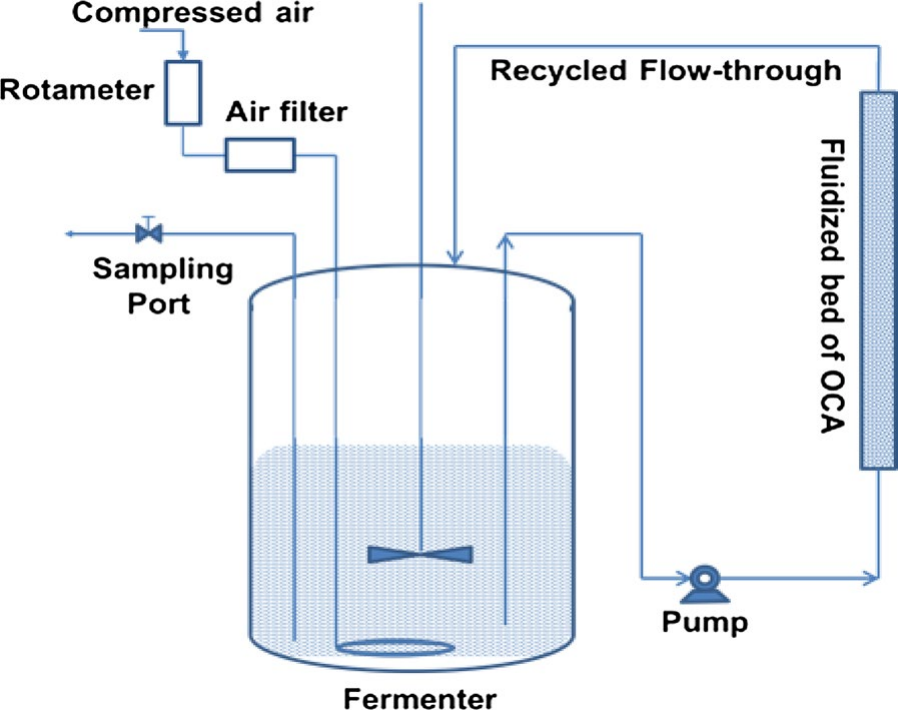
A schematic diagram for microbial oil production and its online capture. The oil production broth from the fermenter was pumped out to continuously fluidize an external OCA bed while the flow-through was recycled back into the fermenter to achieve simultaneous production and online oil capture

**Fig. 9:**
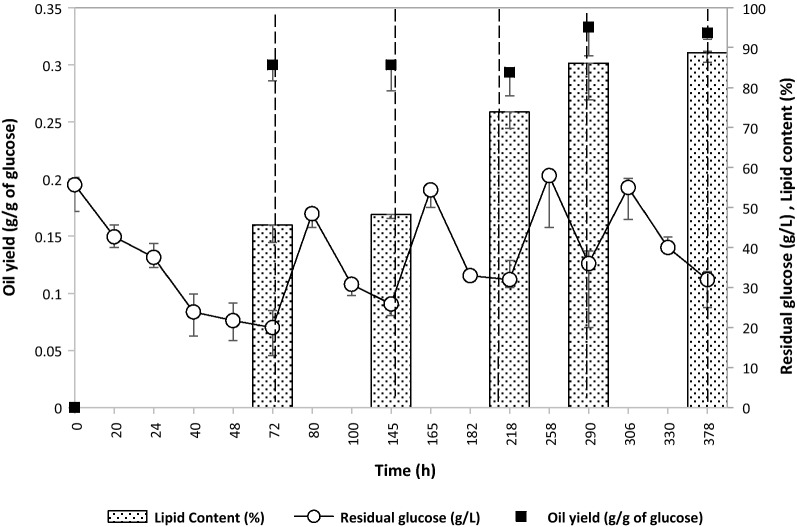
Semi-continuous mode for microbial oil production with an on-line oil capture. The residual glucose, lipid content and oil yield on glucose consumed was studied over 5 cycles of continuous production and on-line oil capture. Each cycle of 72 h was followed with fed-batch addition of glucose (to adjust the glucose concentration to 5% v/v) and regeneration of OCA bed for its reuse in the subsequent cycle. Same cells were used throughout the 378 h operation (5 cycles)

In an endeavor to simplify the extractive production process, the microbial oil production was further tried in a semi-continuous operation mode, with an adsorbent bed in a glass column, fluidized with the oil production media, while forming an external recycle loop with the fermenter (Fig. 8). The OCA bed was replaced (after every 3 days), regenerated, and repeatedly used for duration of as long as 380 h. The lipid content increased up to 89.10% of DCW, while the oil yield was maintained near to the theoretical maximum (Fig. 9).

Figure 8 shows an alternate mode of operation for using OCA in a fluidized bed, forming an external recirculation loop with the fermenter. This mode allows for simultaneous oil production and a concomitant on-line stripping of the produced oil.

**Fig. 10:**
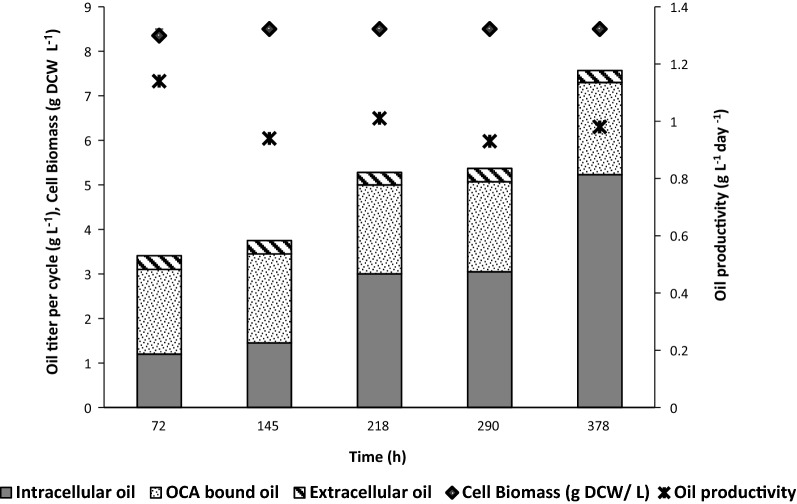
Distribution of microbial oil in a production system with continuous on-line oil capture. Oil production and its distribution in intracellular, extracellular and resin-bound forms in each cycle of continuous production and on-line oil capture was studied

As seen in Fig. 10, there was a gradual increase in the oil titer (g L^−1^) over the period. This increase in the oil titer was in accordance with the increase in intracellular oil content of the cells. Additionally, the bed of OCA was consistently found to capture oil based on its binding capacity, while the extracellular lipid content in the broth was continuously constrained below its saturation limit (viz. about 2 g L^−1^). Therefore, the OCA conceivably caused for the sequestration of oil, thereby pulling the associated flux.

Interestingly, although there was an increase in lipid content (% DCW) over the period, the oil yield over the sugar consumed remained constant throughout the operation and was closer to the theoretical maximum value. This observation signifies the increase in the oil production capacity of the organism about the persisted stress conditions. Moreover, the cell density in the fermenter was found to be constant (at 8.5 g DCW L^−1^) throughout the process, indicating maintenance and arrest of viable cell biomass in a productive stressed phase. Similarly, the oil productivity was maintained at a rate of 1 g L^−1^ day^−1^ after 72 h (Fig. 10) indicating that the system is being continuously reaped (milked) to deliver a specific output. The fluidization allowed for an increased surface area and packaging of differently sized particles inside the column. The column offered a good desorption characteristic as well. Sequential elution of highly resolved individual component lipids from the broth could be achieved with an intermittent adsorptive regeneration process. Periodic regeneration of the bed with the organic solvent caused elution of the adsorbed oil and permitted the reusability of the bed in an integrated mode with the fermentation system. The use of eluting solvents like ethanol and propan-2-ol caused for sterilization in place. However, the productivity in continuous online oil capture mode (1 g L ^−1^day^−1^ or 0.04 g L ^−1^ h^−1^) was lower than that in a two-stage batch production with in situ oil capture (0.166 g L^−1^ h^−1^ or 3.98 g L ^−1^day^−1^) inside the broth (Table 3). Consistent oil productivity per cycle showed a correlation with the constant amount of oil captured and extracellular oil in the broth (Fig. 10). The limitation in increasing oil productivity per cycle is, therefore, associated with the rate of extracellular secretion and adsorption of oil. However, the advancement in this online capture mode lies in effectuating a continuous production and recovery operations of industrial relevance, for milking the yeast cells to deliver oil in natural forms over theoretical glucose conversions. The process is, thus, attractive over the other modes, which require significant investment over the substrates for producing new cells to drive oil production in every batch after undergoing cell-disruptive intracellular lipid extraction.

**Table 3:** Comparison of different strategies used for oil production by oleaginous yeasts using glucose as a C-source

Strain	Culture conditions	Oil productivity (g L^−1^ h^−1^)	Oil yield (*g*_oil_/*g*_glucose consumed_)	References
*Rhodosporidium toruloides* DSMZ 4444	Batch fermentation—N limiting; pO_2_ controlled (with 50% air saturation)	0.15	0.15	[39]
*Saccharomyces cerevisiae* ^E^	Systematic engineering in *S. cerevisiae* for enhanced free fatty acids production; fed-batch addition of glucose to a minimal media	0.104^a^	0.23^a^	[40]
*Y. lipolytica* W29 (ATCC20460)^E^	Metabolic flux pulling and in situ addition of 15% (v/v) dodecane at shake flask scale	0.14^a^	0.20	[15]
*Rhodosporidium toruloides* DSMZ 4444^A^	Cultivation in NaCl-enriched glucose-based media for yeast adaptation	0.075^a^	0.21	[41]
*Rhodosporidium toruloides* ^A^	Shake flask culturing for 96 h; Strain adapted by successive cultivations in increasing concentrations of SCBH	0.106 ± 0.005	0.20	[42]
*Rhodosporidium toruloides* A29	Fed-batch glucose addition to an optimized media to maintain C/N	0.26^a^	0.147^a^	[43]
*Yarrowia lipolytica NCIM 3590*	Two-stage fermentation with 5% glucose as sole carbon and nutrient source	0.066^a^	0.15 ± 0.02	[17]
*Yarrowia lipolytica* Y-4311^E^	Batch cultivation in shaking tubes with 7.5% (v/v) dodecane	0.019^a^	0.25^a^	[29]
*Lipomyces starkeyi* NBRC10381	10% Glucose in nitrogen-limited mineral medium with high inoculum size	0.28^a^	0.20^a^	[44]
*Trichosporon. oleaginosus* ATCC 20509	Fermentation with Excess O_2_ in a media with C/N 75.3	0.14 ± 0.03	0.21	[45]
*Yarrowia lipolytica* ADgm-hi^E^	Engineered cytosolic redox metabolism; Cultured in a high density fed-batch fermentation	1.3	0.269	[46]
*Cryptococcus curvatus* MUCL 29819	Pre-culture followed by batch fermentation with 4% glucose as a sole carbon/nutrient source	0.024^a^	0.088	[13]
*Cutaneotrichosporon oleaginosus* (ATCC 20509)	Sole glucose batch fermentation; in situ enzymatic cell wall hydrolysis for intracellular oil recovery	0.09	nd	[47]
*Lipomyces starkeyi* Ls-D35 strain	Lipid production under low C/N ratio (17.9) by high cell density cultivation	nd	0.13	[48]
*Yarrowia lipolytica* E26E1	Batch fermentation with 16% glucose in minimal medium followed by Switchable solvent extraction (DMCHA, EB, DP) from wet cells	0.099^a^	0.1^a^	[37]
*Yarrowia lipolytica* NCIM 3590	Two-stage microbial oil production with in situ oil capturing agents (OCA); Batch fermentation 72 h; 5% glucose	0.166	0.33	Present study
*Yarrowia lipolytica* NCIM 3590	Oil production with continuous on-line oil capture for 378 h; Fed-batch (5% glucose) addition and OCA regeneration for 5 cycles of 72 h each	0.041	0.33	Present study

*nd* no data reported

^a^Values calculated on the basis of data reported

^E^Genetically modified strain

^A^Adapted strain

Table 3 shows that the oil yields over glucose consumed have always been below theoretical value of 0.33 *g*_oil_/*g*_glucose consumed_. The present study shows considerable advancements over those reported, in terms of the oil production by a non-modified strain of *Yarrowia lipolytica* while using glucose as a sole nutrient source.

### Evaluation of fuel properties based on fatty acid composition of microbial oil

**Fig. 11:**
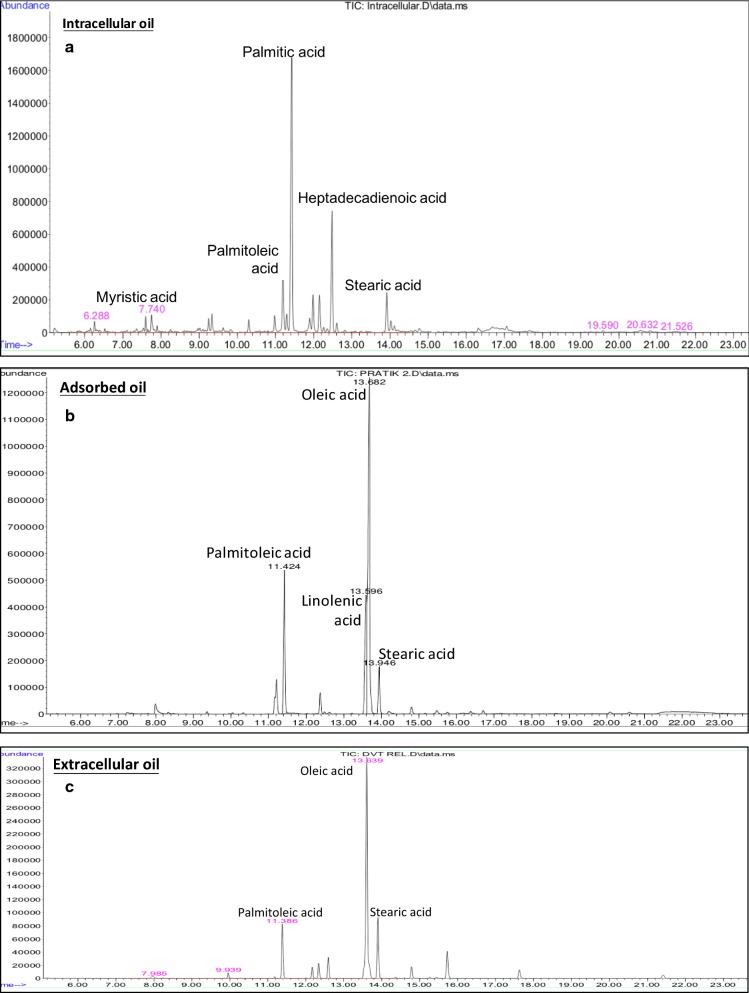
GC–MS characterization of microbial oil. The fatty acid composition profile of the **a** intracellular, **b** adsorbed and **c** extracellular microbial oil has been studied and compared

The GC–MS-based compositional analysis of intracellular microbial oil without in situ OCA showed the presence of myristic acid (C14:0 = 3.23%), Penta decanoic acid (C15:0 = 3.63%), palmitic acid (C16:0 = 43.87%), palmitoleic acid (C16:1 = 9.54%), margaric acid (C17:0 = 0.42%), margaroleic acid (C17:1 = 0.26%), heptadecadienoic acid (C17:2 = 20.56%), stearic acid (C18:0 = 13.9%), oleic acid (C18:1 = 1.74%) and linoleic acid (C18:2 = 2.75%). Whereas, the FAME profile of adsorbent bound microbial oil comprised of palmitic acid (C16:0 = 3.79%), palmitoleic acid (C16:1 = 25.16%), stearic acid (C18:0 = 4.51%), oleic acid (C18:1 = 45.75%) and linoleic acid (C18:2 = 20.79%). Likewise, the un-captured extracellular oil in the broth was found to be predominantly composed of palmitoleic acid (C16:1 = 15.10%), stearic acid (C18:0 = 17.05%) and oleic acid (C18:1 = 50%). Extracellular and adsorbent bound oils both differed from the intracellular oil and showed a notable acyl chain elongation including desaturation in fatty acids (Fig. 11). Nojima et al. also reported extracellular secretion of oleic acid-rich triglycerides when a strain of *Trichosporon* was cultured in a medium with glucose as a sole carbon source [49]. Moreover, the predominance of long-chained unsaturated fatty acids in the extracellular and adsorbent bound oil signify the possibility that the OCA captured most of the extracellular oil secreted.

The biofuel properties of the intracellular and adsorbent bound lipids were assessed based on the fatty acid composition [34] and compared with the national and international standards (Additional file 2) [35]. The increased desaturation in the fatty acid composition greatly influenced its cold filter plugging point (CFPP) and kinetic viscosity (KV) conferring it amenable to the working in colder environments. The chain elongation and apparent lesser free fatty acid composition in the OCA oil helped in lowering the saponification value considerably. Similarly, the density was reduced to 900 kg m^−3^ upon addition of OCA, ascribed to an increased degree of unsaturation. The density of the fuel is required in between 860 and 900 kg m^−3^ since the fuel density affects air–fuel ratio and the energy content in the combustion engine [36]. The degree of unsaturation was also closely related to the iodine value and the oxidative stability of the oil.

Nevertheless, both of these values were in the range set by international standards. The quality of the oil was also found dependent on the content of polyunsaturated fatty acids (PUFA) and monounsaturated fatty acids (MUFA). Higher content of PUFA in OCA oil imparted it lesser viscosity and hence lowered long-chain saturation factor (LCSF). However, a tradeoff between the MUFA and PUFA was essential to have better oxidative stability and combustion characteristics. Lower MUFA in OCA as compared to the native intracellular oil slightly lowered the cetane number 47.28 (min), but it was still in the limits set by ASTM. Although the OCA selectively captured hydrophobic lipids, the calorific values of both the oils were comparable (39 MJ kg^−1^). Thus, based on the conformations with the statutory norms, the OCA captured oil can be speculated as a sustainable feedstock for biofuel production.

## Conclusion

The possibility of concomitant, non-disruptive lipid recovery directly from the broth of a non-modified oleaginous yeast strain of *Yarrowia lipolytica* was examined using an oil capturing agent. The heterogeneous capturing agent demonstrated a high selective binding capacity in the aqueous broth. This strategy eased microbial oil separation from the liquid phase and provided an advantage over the other liquid phase oil extracting agents tried earlier. The simultaneous in situ removal of the produced oil ascertained theoretical conversions and ensured complete substrate consumption by the organism, in every attempted mode of operation. In all these cases, it helped in shelving the saturation and maintaining a systemic equilibrium, while instigating a necessary flux pull towards oil production. Amongst the different ways tried for integrating the process with OCA; the on-line oil capture over an adsorbent bed fluidized with fermentation broth allowed for continuous production and recovery for more than 380 h. The process resulted in lipid content higher than 89% in *Yarrowia lipolytica*, while it was milked for oil continuously. Consistency in the oil yields of 0.33 g per g of glucose demonstrates amenability, and provides a baseline for further scale-up and process intensification for increasing the productivities. The efficiency projected by these values is much higher than various solvent-mediated extraction strategies currently used for microbial oils from oleaginous yeasts [37]. In situ oil removal caused effective integration of the upstream and downstream process, allowing circumvention of energy consuming unit operations. The docile modus operandi sequestered oil in a non-disruptive manner and passively increased substrate flux inside the cells, directing it towards lipid biosynthesis, thereby improvising the oil production. The well-timed capture of the extracellular oil using an OCA prevented its denaturation and re-assimilation. The fatty acid composition of the adsorbed oil was greatly influenced by the addition of OCA and showed comparable differences with its intracellular counterparts without OCA. Biofuel properties estimated based on these improved fatty acid compositions illustrate smoother engine operations, reduced emissions, and better performance at lower temperatures. The combustion characteristics revealed energy content of 39 MJ kg^−1^. These values were competent to the jatropha and other plant-based oils currently used as feedstocks for biodiesel production [35]. Hence, the demand for an alternative sustainable energy feedstock can be calmed to a great extent using microbial oils produced by this route. A continuous ‘Microbial Oil Factory’ operating at theoretical efficiencies, thus, realizes a conceivable calorific valorization of hydrogen deficient lignocellulosic biomass through a greener route, while climbing fewer steps on H_eff_/C staircase.

## Methods

### Materials

#### Oleaginous yeast strain and culture medium

Stock culture of *Yarrowia lipolytica* NCIM 3590 strain procured from National Collection of Industrial Microorganisms (NCIM)-National Chemical Laboratory, Pune, India was maintained at − 80 °C in MGYP (Malt Extract, Glucose, Yeast extract, and Peptone) medium with 40% (v/v) glycerol. The stock was revived in a 50-mL MGYP medium and incubated at 28 °C for 48 h. This culture was used for pre-inoculum development in the MGYP growth medium with composition per liter: 3-g malt extract, 20-g glucose, 3-g yeast extract, 5-g peptone.

#### Two-stage process for microbial oil production

##### Growth phase

The seed culture was transferred to the experimental flasks to have an inoculum load of 10% (v/v). The flasks were incubated at 28 °C at 200 rpm for 48 h. The growth phase was carried out in a chemostat mode to obtain the desired cell density, as described earlier [17]. The moisture content of the wet cell biomass obtained after centrifugation of the broth was calculated on a Moisture Analyzer (METTLER TOLEDO, HE53) and the dry cell weight (DCW) was estimated accordingly.

##### Oil production phase

The oil production media with glucose (5%) as the sole carbon and nutrient source was transferred to a 5-L fermenter (BIOSTAT B plus, Sartorius, Germany) having a working volume of 2 L along with the obtained yeast cells, achieving a cell density of 25 g L^−1^ (i.e., 5 g DCW L^−1^). The fermenter was operated at 200 rpm and 28 °C for 72 h with aeration of 1 vvm. 10 mL of broth was harvested after every 24 h, centrifuged at 13,000*g* at 14 °C for 2 min, and the supernatant was analyzed for residual glucose and extracellular oil. The cell pellet was weighed for determining the concentration of cell biomass and further subjected to intracellular oil extraction.

#### Analysis of sugar and estimation of glucose uptake rate

Glucose concentration was determined by high-performance liquid chromatography (HPLC, Agilent series 1200, Japan) system equipped with a refractive index detector. The HPLC column used was Aminex 87H (Bio-Rad, Hercules, CA) and elution was carried out with 5-mM sulphuric acid as the mobile phase at a flow rate of 0.6 mL min^−1^. The column temperature was maintained at 50 °C. The standard calibration plot for glucose was used for the quantitative determination of sugar in unknown samples.

Consumption of glucose was, thus, estimated, and the Glucose uptake rate was calculated by the formula (as shown in Eq. 1)

1 $${\text{Glucose uptake rate }}({\text{g L}}^{ - 1} {\text{h}}^{ - 1} ) = \left( {{\text{Glucose consumed}}\left( {{\text{g}}\;{\text{L}}^{ - 1} } \right)} \right)/\left( {{\text{Time }}\left( {\text{h}} \right)} \right).$$

#### Oil capture using hydrophobic OCA

##### In situ oil capture

The oil capturing agents were introduced into the oil production broth at the onset of the fermentation, to achieve a concomitant production and a simultaneous in situ oil capture inside the broth. Initial studies with in situ OCA at shake flask level were carried out by adding 1-g SEPABEADS™ SP70 (obtained from Mitsubishi Chemical Corporation, India), as an oil capturing agents (OCA) to the oil production medium along with the inoculation of cell biomass obtained from the growth phase cells.

##### Ex situ oil capture

100 g of oil capturing agents, having an oil binding capacity of 0.122 g g^−1^, was contacted to the oil production broth for 30 min, post-production; (i.e., after 72 h of oil production) in the shake flask at 200 rpm. The OCA was added in excess considering the oil production capacity and the binding capacity of the resins. After contacting the broth with OCA for 12 h, the oleaginous cells and OCA were separated by filtration. This was followed by centrifugal separation of the aqueous supernatant. The oleaginous cells, OCA, and the aqueous phase were further subjected to different analytical procedures for oil estimation.

### Glucose uptake studies

Cells grown in the chemostat were centrifuged and re-suspended in varying concentrations of glucose (1%–6%) and incubated at 28 °C under shake flask conditions. The rate of glucose uptake was monitored continuously for 3 days. This was also compared with a similar set containing 1-g SEPABEADS™ SP70 (obtained from Mitsubishi Chemical Corporation, India), as an oil capturing agents (OCA) for the same period. Oil produced by the cells was also quantified and production rate calculated.

### Extraction of microbial oil from oleaginous yeast and estimation of the oil production rate

Microbial oil from oleaginous yeast cells was extracted in the form of ‘Intracellular Lipid,’ ‘Extracellular lipid’ in the broth, and the ‘OCA bound oil.’ The wet cell biomass, obtained as a pellet after centrifugation of the oil production broth, was used for intracellular lipid extraction using a solvent mixture 1:1 (v/v cells). The solvent mixture was prepared using equal volumes of propan-2-ol and chloroform, for cell disruption and complete intracellular lipid extraction [13]. The aqueous supernatant obtained after centrifugation of the oil production broth was also subjected to solvent extraction with chloroform in a ratio of 1:1 (v/v), so as to obtain extracellular oil in the organic phase. The oil adsorbed on the resin beads was obtained after desorption of the resins with 2 column/bed volumes of a solvent mixture of propan-2-ol and chloroform. Extraction was repeated to ensure complete lipid extraction. The total microbial oil, thus, obtained after distillation of the solvent organic phases from each extraction was quantified gravimetrically.

Extractive solvents used for analysis and extraction were of analytical grade or chromatographically pure and were obtained from SD Fine Chemicals Ltd., India. Oil production rate or productivity (g L^−1^ h^−1^) was calculated, as shown in Eq. (2).

2 $${\text{Oil production rate}}/p{\text{roductivity }}({\text{g L}}^{ - 1} {\text{h}}^{ - 1} ) = \left( {{\text{Oil titer}}\left( {{\text{g}}\;{\text{L}}^{ - 1} } \right)} \right)/\left( {{\text{Time }}\left( {\text{h}} \right)} \right).$$

### Two-stage microbial oil production with in situ oil capture using an oil capturing agent (OCA)

Oil production with OCA was carried out in a 5-L jacketed glass fermenter (BioFlo^®^ 120, Eppendorf, India), with 2-L working volume of oil production broth comprising 50 g L^−1^ glucose solution. The oil production was carried out for 72 h at 28 °C and 200 rpm, while the aeration was maintained at 1 vvm. Regenerated oil capturing agent (OCA), SEPABEADS™ SP70, was added to the fermenter at the onset of fermentation. A resin load of more than 1% (i.e., 25 g of SEPABEADS™ SP70) was considered based on the oil binding capacity (0.14 g g^−1^) and oil saturation concentration of 3 g L^−1^ of the aqueous phase. The yeast cell mass was added to the fermenter, to have an effective cell load of around 5 g DCW L^−1^ in the broth. The regeneration of the resins was carried out before their addition to the broth, with repeated washes of propan-2-ol followed by at least three washes with sterile water, to ensure activation of the binding sites with complete removal of bound moieties and the eluting solvent propan-2-ol. The regeneration procedure also helped in sterilizing the adsorbent resin beads before their use.

### Continuous on-line capture of microbial oil

A 10-cm glass column (5 cm internal diameter) packed with 25 g (117 cm^3^) of the OCA (packed bed height = 6 cm) was fluidized up to 7.1 cm with the oil production broth at 10 mL min^−1^ flowrate from the bottom of the column. The flow-through from the column containing viable cells was recirculated back to the fermenter. The adsorbed column was replaced after 3 days with a regenerated column. Regeneration of the adsorbent bed for its reuse and recovery of the adsorbed oil was carried out by elution with 1 column volume of propan-2-ol and chloroform (1:1 v/v) followed by elution with 3 column volumes of sterile water. The organic phase eluent was distilled to obtain oil, which was quantified gravimetrically. In this semi-continuous mode of operation, switching of the columns was accompanied with glucose addition, to maintain a requisite glucose concentration of 5% in the oil production broth.

### Sudan black staining

The broth samples withdrawn at periodic intervals from the oil production phase of the two-stage process were stained using Sudan Black B stain and observed at 100× magnification under a light microscope as described earlier [21].

### Fluorescent microscopic analysis of extracellular oil using Nile red dye staining

250 mg of Nile red dye powder procured from the Sigma Aldrich, USA, was dissolved in 1 mL of acetone and used as a stock solution. A 100× dilution of the stock was used to stain the broth samples from different phases. Microscopic analysis of the samples on the slide as well as in a deep-well plate was carried out on Lionheart FX Automated Microscope (BioTek Instruments India (P) Limited) equipped with 16-bit grayscale Sony CMOS camera. Microscopic examination was done using 20×/40× Olympus Plan Fluorite Phase objectives having numerical aperture (NA)—0.45, depth of field—2.9 µm and at a working distance—6.4 to 7.6 mm. Fluorescence analysis was carried at 531/40 nm excitation and 593/40 nm emission wavelengths with LED-523 nm using RFP filter cube. Auto expose and Autofocus feature of Gen5 3.0 software was used, wherein the software automatically selects the camera gain depending on the quality of the image. The same software was used to capture the images in phase contrast, fluorescence channel, and process them further to overlay these images.

### FTIR characterization

Fourier transforms infrared (FTIR) spectra of the oils, and intact cells were obtained using FTIR-8400S Shimadzu spectrometer. The sample was directly placed on the sample holder cell for IR determination. Cells from different phases were washed thrice with distilled water to avoid medium contamination. 2 mL of the cell suspensions was dried in a vacuum oven at 35 °C for about 5 h to ensure removal of the excess water. FTIR transmission spectra were acquired between 4000 and 400 cm^−1^, after spectral corrections for residual water vapor absorption.

### LC–MS analysis

Intracellular microbial oil and oil captured on the OCA were analyzed for its components on the HPLC, using Methanol: Water (1% TFA) (90:10 v/v) and dichloromethane gradient elution method at 1 mL min^−1^ flow rate on a C-18 column for 40 min with a column temperature of 30 °C, to effectively separate out the individual components of the oil as Free fatty acid, glycerides (MAG, DAG, TAG), Phospholipids, etc. The peaks, as seen in the chromatogram, were collected as individual fractions for their prospective analysis on the MS system. The fractionated samples were dried in a vacuum oven and re-dissolved in MS grade acetonitrile, to make a concentration of 10 ppm, for further analysis. LC–MS analysis with Q-TOF was firstly performed in a ‘full scan mode’ in the MS range 100–1500 *m/z* with a direct infusion of 1-µL fractionated samples. Carrier Mobile phase used was ACN: Water (1% Formic acid) (50:50 v/v). The analysis was performed with ESI in both positive and negative modes of ionization for each fraction. Structural elucidation based on LC–MS spectra was carried out using online lipid structure database LIPID MAPS^®^ Lipidomics Gateway Structure Database (LMSD) http://www.lipidmaps.org.

### GC–MS analysis of the microbial oil and evaluation of fuel properties

Analysis of the fatty acid composition was performed using GC–MS after derivatization of the oil samples to fatty acid methyl ester (FAME) by the protocol optimized by Shantha and Napolitano [38]. The analysis was performed on Agilent Technology 7890A GC having 5975C MS system, equipped with an HP5MS capillary column (30 m × 0.20 mm, 0.33-μm coating thickness). The FAMEs were ascertained by (NIST11.L database) library search reports. The biofuel properties were estimated by previously reported empirical equations [34] and were evaluated for their compliance with the prescribed standards set by American Society for Testing and Materials (ASTM D6751), European (EN 14214) and Indian Standard (IS 15607).

## Supplementary information

**Additional file 1.** A: LC MS characterization of intracellular microbial oil. B: LC MS characterization of of the oil captured on OCA.

**Additional file 2.** Biofuel properties of microbial oil based on the fatty acid composition.

## Data Availability

All data generated or analyzed during this study are included in this published article and its additional files.

## Abbreviations

ACN: acetonitrile
ASTM: American Society for Testing and Materials
CFPP: cold filter plugging point
CSTR: Continuous Stirred Tank Reactor
DAG: di acyl glycerol
DCW: dry cell weight
FA: fatty acid
FTIR: Fourier-transform infrared spectroscopy
H_eff_/C: effective hydrogen-to-carbon ratio
KV: kinematic viscosity
LC–MS: liquid chromatography–mass spectrometer
LCSF: long-chain saturation factor
GC MS: gas chromatography–mass spectrometer
FAME: fatty acid methyl ester
LD: lipid droplets
MGYP: malt extract, glucose, yeast extract, peptone
MUFA: mono-unsaturated fatty acid
OCA: oil capturing agent
PA: phosphatidic acid
PUFA: poly-unsaturated fatty acid
Q-TOF: quadrupole time-of-flight mass spectrometry
TAG: tri-acyl glycerol
TFA: trifluroacetic acid
ESI: electrospray ionization
*m/z*: ratio of mass to charge
