# Supplementary material for: Simultaneous lipid biosynthesis and recovery for oleaginous yeast Yarrowia lipolytica
Source: Biotechnol Biofuels. 2019 Oct 8;12:237. doi: 10.1186/s13068-019-1576-7 (PMC6781333; doi:10.1186/s13068-019-1576-7)

Additional files

Additional file 1A: LC MS characterization of intracellular microbial oil

**Additional file 1B**:

LC MS characterization of of the oil captured on OCA


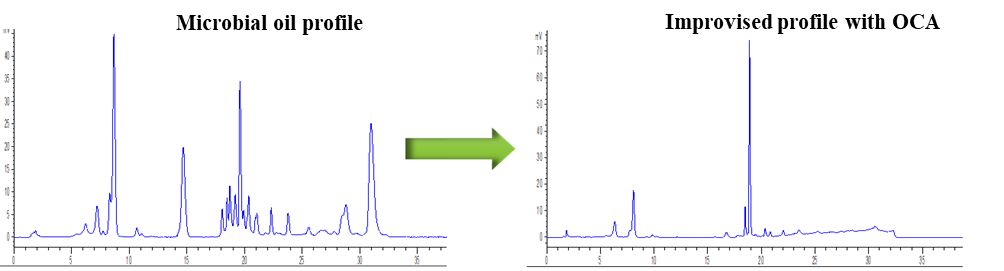

Supplement: Supplementary file 1 — Additional file 1. A: LC MS characterization of intracellular microbial oil. B: LC MS characterization of of the oil captured on OCA. [file 13068_2019_1576_MOESM1_ESM.docx]
