# Supplementary material for: Simultaneous lipid biosynthesis and recovery for oleaginous yeast Yarrowia lipolytica
Source: Biotechnol Biofuels. 2019 Oct 8;12:237. doi: 10.1186/s13068-019-1576-7 (PMC6781333; doi:10.1186/s13068-019-1576-7)
Supplement: Supplementary file 2 — Additional file 2. Biofuel properties of microbial oil based on the fatty acid composition. [file 13068_2019_1576_MOESM2_ESM.docx]

Additional file 2:

Biofuel properties of microbial oil based on the fatty acid composition

| **Value** | **Prescribed range** | **Intracellular oil**  **(Without OCA)** | **Oil adsorbed on OCA** | **Remarks/ Conclusion** |
| --- | --- | --- | --- | --- |
| Saponification value mg KOH/g oil | 180-200 mg KOH/g oil  (ISO 3657-2002) | 214.03 | 204.73 | - FFA’s in the oil readily undergo saponification, hence the saponification value of the OCA oil was less - Moreover, as seen from the fatty acid profile chain elongation was observed with OCA, therefore lowering the saponification value in case of OCA bound oil |
| Iodine value mgI_2_/100 g | 120 mg I_2_/100 g  (EN 14214 ) | 66.76 | 100.71 | - The experimental and empirically derived values of IV were comparable for each of the oils - The unsaturation in the OCA bound oil was responsible for higher IV - However, these values were still within the prescribed range below the maximum of 120 mg I_2_/100 g |
| Cetane number (min) | 47  (ASTMD6751)  51 (EU14214 & IS 15607) | 54.78 | 47.28 | - The ignition characteristics of both the oils were comparable to each other - However, the unsaturation in the OCA bound oil reduces its cetane number and thereby the fuel efficiency rendering lower H_eff_/C ratio |
| Degree of Unsaturation | Not specified | 58.16 | 112.49 | - Degree of unsaturation of OCA bound oil is very high considering the higher content of unsaturated fatty acids in their profile - Although, the oxidation stability is very low in this case |
| Long chain saturation factor | ≤5 (%wt) Microbial oil 2.8/2.9 | 11.34 | 2.63 | - The lower LCSF value of OCA oil suggests PUFA, which imparts low viscosity as compared to its counterpart which contains short chain saturated fatty acids |
| Cold filter plugging point ($℃)$ | Around -6 to -7 | 22.26 | -7.48 | - Higher PUFA in OCA oil confers it lower CFPP making it amenable to working at cold temperatures |
| High heating / Calorific value MJ/kg | Both ASTM D6751 and EN 14214 standards do not have any specification for HHV | 39.65 | 39.53 | - Although the OCA selectively adsorbs to specific more hydrophobic lipids, the calorific values of both the oils are more or less identical |
| Kinematic viscosity mm^2^/s | 1.9–6.0 mm2/s  (ASTM D 445)  3.5–5.0 mm2/s.  (EN ISO 3104) | 4.35 | 2.22 | - The KV of both the oils fall in the prescribed limit - The unsaturation in OCA confers lower viscosity and operability of oil in cold temperatures |
| Density | 860–900 kg m−3 at 15 °C (EN 14214 ) | 0.95 | 0.9 | - The density of both the oils calculated theoretically and experimentally was closer to each other irrespective of their fatty acid profiles. - This would be due to the trapped moisture in the oils which accounts for certain weight, rendering the density closer to unity |
| Oxidative stability (h) | ≥6 h (EU 14214 & IS 15607)IS 15607) | 45.47 | 8.26 | - The oxidative stability of the OCA oil was decreased almost five fold owing to higher PUFA leading to greater oxidative potential of these oils rendering them susceptible to rancidity |
